# Supplementary material for: Classroom effects of a preventive behavioral management program: A pragmatic cluster-randomized trial of Good Behavior Game
Source: PLOS Ment Health. 2025 Dec 23;2(12):e0000487. doi: 10.1371/journal.pmen.0000487 (PMC12798361; doi:10.1371/journal.pmen.0000487)
Supplement: S1 CONSORT Checklist — CONSORT, Consolidated Standards of Reporting Trials. © 2025 Hopewell et al. This is an Open Access article distributed under the terms of the Creative Commons Attribution License (https://creativecommons.org/licenses/by/4.0/), which permits unrestricted use, distribution, and reproduction in any medium, provided the original work is properly cited. (DOCX) [file pmen.0000487.s001.docx]

**S1 CONSORT Checklist for Classroom effects of a preventive behavioral management program: A pragmatic cluster-randomized trial of Good Behavior Game**

|  | Section/topic | No | CONSORT 2025 checklist item description | Reported on page no. |
| --- | --- | --- | --- | --- |
|  | **Title and abstract** | | |  |
|  | Title and structured abstract | 1a | Identification as a cluster-randomised trial* | 1 |
|  |  | 1b | Structured summary of the trial design, methods, results, and conclusions | 2 |
|  | **Open science** | | |  |
|  | Trial registration | 2 | Name of trial registry, identifying number (with URL) and date of registration | 7 |
|  | Protocol and statistical analysis plan | 3 | Where the trial protocol and statistical analysis plan can be accessed | 7, 34 |
|  | Data sharing | 4 | Where and how the individual de-identified participant data (including data dictionary), statistical code and any other materials can be accessed | 17, See Data availability |
|  | Funding and conflicts of interest | 5a | Sources of funding and other support (eg, supply of drugs), and role of funders in the design, conduct, analysis and reporting of the trial | See Funding |
|  |  | 5b | Financial and other conflicts of interest of the manuscript authors | See Competing interests |
|  | **Introduction** | | |  |
|  | Background and rationale | 6 | Scientific background and rationale. Rationale for using a cluster design* | 3-8 |
|  | Objectives | 7 | Specific objectives related to benefits and harms. Whether objectives pertain to the cluster level, the  individual participant level, or both. If pre-specified, how the intervention was hypothesised  to work* | 6-8 |
|  | **Methods** | | |  |
|  | Patient and public involvement | 8 | Details of patient or public involvement in the design, conduct and reporting of the trial | 7-8 |
|  | Trial design | 9 | Description of trial design including type of trial (eg, parallel group, crossover), allocation ratio, and framework (eg, superiority, equivalence, non-inferiority, exploratory). Definition of cluster and description of how the design features apply to the clusters* | 7-9 |
|  | Changes to trial protocol | 10 | Important changes to the trial after it commenced including any outcomes or analyses that were not prespecified, with reason | 8-9 |
|  | Trial setting | 11 | Settings (eg, community, hospital) and locations (eg, countries, sites) where the trial was conducted | 7-8 |
|  | Eligibility criteria | 12a | Eligibility criteria for participants/clusters* | 8-9 |
|  |  | 12b | If applicable, eligibility criteria for sites and for individuals delivering the interventions (eg, surgeons, physiotherapists) | 8-11 |
|  |  | 12c | Eligibility criteria should be explicitly framed to show the degree to which they include typical participants and/or, where applicable, typical providers (eg, nurses), institutions (eg, hospitals), communities (or localities eg, towns) and settings of care (eg, different healthcare financing systems)* | 6-8 |
|  | Intervention and comparator | 13a | Intervention and comparator with sufficient details to allow replication. If relevant, where additional materials describing the intervention and comparator (eg, intervention manual) can be accessed. Whether interventions pertain to the cluster level, the individual participant level, or both* | 9-11 |
|  |  | 13b | Describe extra resources added to (or resources removed from) usual settings in order to implement intervention. Indicate if efforts were made to standardise the intervention or if the intervention and its delivery were allowed to vary between participants, practitioners, or study sites* | 9-11 |
|  |  | 13c | Describe the comparator in similar detail to the intervention* | 11 |
|  | Outcomes | 14a | Prespecified primary and secondary outcomes, including the specific measurement variable (eg, systolic blood pressure), analysis metric (eg, change from baseline, final value, time to event), method of aggregation (eg, median, proportion), and time point for each outcome. Whether outcome measures pertain to the cluster level, the individual participant level, or both* | 11-17 |
|  |  | 14b | Explain why the chosen outcomes and, when relevant, the length of follow-up are considered important to those who will use the results of the trial* | 6-8, 10 |
|  | Harms | 15 | How harms were defined and assessed (eg, systematically, non-systematically) | 11-12 |
|  | Sample size | 16a | How sample size was determined, including all assumptions supporting the sample size calculation. Method of calculation, number of clusters(s) (and whether equal or unequal cluster sizes are assumed), cluster size, a coefficient of intracluster correlation (ICC or k), and an indication of its uncertainty* | 9, study protocol |
|  |  | 16b | Explanation of any interim analyses and stopping guidelines | NA |
|  |  | 16c | If calculated using the smallest difference considered important by the target decision maker audience (the minimally important difference) then report where this difference was obtained* | NA |
|  | Randomisation: |  |  |  |
|  | Sequence generation | 17a | Who generated the random allocation sequence and the method used | 7, study protocol |
|  |  | 17b | Type of randomisation and details of any restriction (eg, stratification, blocking and block size) | 7 |
|  | Allocation concealment mechanism | 18 | Mechanism used to implement the random allocation sequence (eg, central computer/telephone; sequentially numbered, opaque, sealed containers), describing any steps to conceal the sequence until interventions were assigned. Specification that allocation was based on clusters rather than individuals and whether allocation concealment (if any) was at the cluster level, the individual participant level, or both* | 7-8, study protocol |
|  | Implementation | 19a | Whether the personnel who enrolled and those who assigned participants to the interventions had access to the random allocation sequence | 7 |
|  |  | 19b | Who generated the random allocation sequence, who enrolled clusters, and who assigned clusters to  interventions* | 7-9 |
|  |  | 19c | Mechanism by which individual participants were included in clusters for the purposes of the trial (such as complete enumeration, random sampling)* | Study protocol |
|  |  | 19d | From whom consent was sought (representatives of the cluster, or individual cluster members, or both) and whether consent was sought before or after randomisation* | 8, 17 |
|  | Blinding | 20a | Who was blinded after assignment to interventions (eg, participants, care providers, outcome assessors, data analysts) | 13 |
|  |  | 20b | If blinded, how blinding was achieved and description of the similarity of interventions | 13 |
|  |  | 20c | If blinding was not done, or was not possible, explain why* | 13 |
|  | Statistical methods | 21a | Statistical methods used to compare groups for primary and secondary outcomes, including harms. How clustering was taken into account* | 16-17 |
|  |  | 21b | Definition of who is included in each analysis (eg, all randomised participants), and in which group | 9 |
|  |  | 21c | How missing data were handled in the analysis | 19 |
|  |  | 21d | Methods for any additional analyses (eg, subgroup and sensitivity analyses), distinguishing prespecified from post hoc | 16-17 |
|  | **Results** | | |  |
|  | Participant flow, including flow diagram | 22a | For each group, the numbers of clusters that were randomly assigned, received intended treatment, and were analysed for the primary outcome. Where possible, the number approached, screened, and eligible prior to random assignment, with reasons for non-enrolment* | 9 |
|  |  | 22b | For each group, losses and exclusions for both clusters and individual cluster members, together with reasons* | 9 |
|  | Recruitment | 23a | Dates defining the periods of recruitment and follow-up for outcomes of benefits and harms | 8-9 |
|  |  | 23b | If relevant, why the trial ended or was stopped | NA |
|  | Intervention and comparator delivery | 24a | Intervention and comparator as they were actually administered (eg, where appropriate, who delivered the intervention/comparator, how participants adhered, whether they were delivered as intended (fidelity)) | 9-11, 16, 20 |
|  |  | 24b | Concomitant care received during the trial for each group | 11 |
|  | Baseline data | 25 | Baseline characteristics for the individual and cluster levels as applicable for each group. Include socioeconomic variables where applicable* | 18-20 |
|  | Numbers analysed,  outcomes and estimation | 26 | For each primary and secondary outcome, by group:  ● the number of participants/clusters included in the analysis  ● the number of participants/clusters with available data at the outcome time point  ● result for each group, and the estimated effect size and its precision (such as 95% confidence interval)  ● for binary outcomes, presentation of both absolute and relative effect size | 9, 20-24 |
|  | Harms | 27 | All harms or unintended events in each group | NA |
|  | Ancillary analyses | 28 | Any other analyses performed, including subgroup and sensitivity analyses, distinguishing pre-specified from post hoc | 16-17, study protocol |
|  | **Discussion** | | |  |
|  | Interpretation | 29 | Interpretation consistent with results, balancing benefits and harms, and considering other relevant evidence | 24-33 |
|  | Limitations | 30a | Trial limitations, addressing sources of potential bias, imprecision, generalisability, and, if relevant, multiplicity of analyses. Generalisability to clusters and/or individual participants (as relevant)* | 24-33 |
|  |  | 30b | Describe key aspects of the setting which determined the trial results. Discuss possible differences in other settings where clinical traditions, health service organisation, staffing, or resources may vary from those of the trial* | 24-33 |

Citation: Hopewell S, Chan AW, Collins GS, Hróbjartsson A, Moher D, Schulz KF, et al. CONSORT 2025 Statement: updated guideline for reporting randomised trials. BMJ. 2025; 388:e081123. <https://dx.doi.org/10.1136/bmj-2024-081123>
© 2025 Hopewell et al. This is an Open Access article distributed under the terms of the Creative Commons Attribution License (<https://creativecommons.org/licenses/by/4.0/>), which permits unrestricted use, distribution, and reproduction in any medium, provided the original work is properly cited.

We strongly recommend reading this statement in conjunction with the CONSORT 2025 Explanation and Elaboration and/or the CONSORT 2025 Expanded Checklist for important clarifications on all the items. We also recommend reading relevant CONSORT extensions. See [www.consort-spirit.org](http://www.consort-spirit.org).

*Indicates a modified or added item. This checklist has been modified using CONSORT Cluster, CONSORT Pragmatic Trials, and CONSORT-SPI. See main paper for references.
