# Supplementary material for: Classroom effects of a preventive behavioral management program: A pragmatic cluster-randomized trial of Good Behavior Game
Source: PLOS Ment Health. 2025 Dec 23;2(12):e0000487. doi: 10.1371/journal.pmen.0000487 (PMC12798361; doi:10.1371/journal.pmen.0000487)
Supplement: Attachment — Submitted filename: Response to Reviewers.docx [file pmen.0000487.s003.docx]

# Response to Reviewers for “Classroom effects of a preventive behavioral management program: A pragmatic cluster-randomized trial of Good Behavior Game”

We are thankful to the reviewers for their decidedly well-informed, insightful and constructive comments, as well as the opportunity to improve our paper. In this letter we list each point of constructive feedback raised by the reviewers and how we have incorporated them into a new revision of the manuscript with tracked changes.

**Reviewer 1**

*Comment:* Article is well written. Accept.The study has been conducted well and manuscript is written flawlessly. Need for the study, methodology and results are understandable and scientific. Discussion and limitations are written adequately. Hence the manuscript can be considered for publication.

*Response:* We are grateful for the positive feedback.

**Reviewer 2**

*Comment:* Introduction part: Please condense history and focus on rationale because it is too lengthy and repetitive.

*Response:* We appreciate the feedback. The part concerning history in the Introduction has been considerably condensed.

*Comment:* Challenges paragraph: Also merge ideas into one cohesive argument, check it because redundant phrasing

*Response:* The part concerning challenges has now been condensed and reorganized, so that examples flow from a single argument.

*Comment:* Discussion parts: Streamline and strengthen interpretive synthesis due to repetitive cautious tone

*Response:* We have done work to improve wording and consistency and removing some redundancy throughout the Discussion. We have also revised sections pertaining to interpretive synthesis in line with this and related comments.

*Comment:* Limitation parts: It is redundant sample size discussion, condense to one precise paragraph (“The study’s small number of clusters limits statistical power and generalizability, though stratified randomization helped mitigate potential imbalance”).

*Response:* We are grateful for the suggestion which has been used to revise and condense the discussion regarding sample size in the Limitations section, together with another reviewer’s suggestion.

*Comment:* Conclusion: Please replace with more scholarly terms ("imprecise", "low precision")
"As effects were relatively uncertain, evidence on specific outcomes and their trajectories are inconclusive...") => "Although estimates were imprecise due to sample constraints, findings were directionally consistent with prior evidence, lending cautious support to GBG’s applicability under pragmatic conditions”.

*Response:* We thank you for the suggested wording which has been directly incorporated to revise the Conclusion, and the recommended terms have been incorporated throughout the paper for increased consistency.

*Comment:* add one concise summary table of key Bayesian results for clarity, that would be helpful

*Response:* A new Table 3 has been added to summarize key estimates of change in a concise table.

*Comment:* Shorten figure captions that currently restate results in full sentences (simplify and align with journal style).

*Response:* Thank you for the feedback. We have now added clarifying text to summarize general elements of the figures and shortened all captions and legends associated with figures under Results.

**Reviewer 3**

*Comment:* please see the attached document for comments. also,the manuscript needs to be language edited and formatted making sure that it is written in the past tense and not future tense as it is a report from a completed study.

*Response:* Thank you for the detailed feedback. We have changed to past tense in the relevant places, along with correcting or clarifying revisions according to each comment in the attached copy.

**Reviewer 4**

*Comment:* 1. The study is underpowered due to having only five randomized clusters (schools). While the authors acknowledge this, it needs stronger, clearer articulation:
a. Emphasize that the small number of clusters directly limits inferential certainty and ability to detect group differences.

*Response:* Thank you for the feedback, this has now been directly incorporated into the limitations.

*Comment*: b. Explicitly state that directional trends should not be interpreted as evidence of effectiveness, only feasibility and plausibility.

*Response:* This has now been stated in the conclusions in a more direct fashion.

*Comment:* c. Consider adding a simulation-based or literature-based justification of the detectable effect size under the current design.

*Response:* While we think a specific simulation would have been preferable, which we now state explicitly, we have now added an earlier simulation-based study which we hope will give the reader some indication of how our statistical methods fare under similar sample sizes.

*Comment:* 2. The manuscript states this was a “highly pragmatic” trial, but does not clearly describe:
a. How pragmatic decisions influenced implementation fidelity and support.
b. Which components differed from “standard GBG efficacy implementation models.

*Response:* Thank you for the opportunity to clarify. We have added some discussion where we attempt to clarify some key features that makes this trial pragmatic, but also why it is difficult to assess how trial conditions and implementation differs from standard due to lower adherence to reporting standards and/or less rigorous reporting standards for these factors in earlier trials. We have also added a sentence to clarify that we have likely not captured reactive adaptations, which may constitute an important difference in a pragmatic trial.

*Comment:* 3. The manuscript frames results as “tentative support.” This is reasonable, but certain phrasing throughout reads as overly optimistic given overlapping HPD intervals (e.g., “Effects were largely in a favorable direction” and “This study lends tentative support for cross-cultural transportation”). I recommend strengthening the language distinguishing statistical uncertainty vs. practical plausibility, especially in the Discussion and Conclusion.

*Response:* Thank you for the valuable feedback. The manuscript has been revised throughout for more consistent and precise language to frame results in a balanced manner.

*Comment:* 4. Teacher-rated conduct problems and climate are not blinded, and intervention teachers were primed through training. This creates risk of expectancy and reporting bias. This must be acknowledged more centrally as a study limitation, particularly because observer outcomes carry “n = 20 classrooms” and minimal rater–calibration data.

*Response:* The methodology concerning raters and their corresponding limitations have been added as a central issue under Limitations.

*Comment:* 5. The paper notes that 80% of teachers were certified, but does not analyze outcomes by certification status. Even a descriptive comparison would strengthen interpretability.

*Response:* A Table 4 has now been added to present descriptive statistics based on certification status, along with some related revisions in the Discussion.

*Comment:* 6. Phrases like “effects were uncertain” and “consistent with earlier research” in the Abstract are slightly contradictory. Please clarify that consistency refers to direction, not magnitude or certainty.

*Response:* Thank you for the opportunity to clarify. These parts are now revised in the Abstract in line with the comment and similar changes based on feedback.
